# Recent Advances in the Mechanisms of Postoperative Neurocognitive Dysfunction: A Narrative Review

**DOI:** 10.3390/biomedicines13010115

**Published:** 2025-01-07

**Authors:** Tingting Wang, Xin Huang, Shujun Sun, Yafeng Wang, Linlin Han, Tao Zhang, Tianhao Zhang, Xiangdong Chen

**Affiliations:** 1 Department of Anesthesiology, Union Hospital, Tongji Medical College, Huazhong University of Science and Technology, Wuhan 430022, China; wangtt201307@163.com (T.W.); xinhuang123abc@163.com (X.H.); sunshujun_hust@foxmail.com (S.S.); wangyafengaaa@163.com (Y.W.); lynn0624@126.com (L.H.); 2019xh0150@hust.edu.cn (T.Z.); d202482175@hust.edu.cn (T.Z.); 2 Institute of Anesthesia and Critical Care Medicine, Union Hospital, Tongji Medical College, Huazhong University of Science and Technology, Wuhan 430022, China; 3 Key Laboratory of Anesthesiology and Resuscitation, Huazhong University of Science and Technology, Ministry of Education, Wuhan 430022, China

**Keywords:** postoperative cognitive dysfunction, neuroinflammation, neurotransmitter imbalance, oxidative stress, anesthetic agents, mitochondrial dysfunction, white matter lesions, encephalocele, sepsis-associated encephalopathy

## Abstract

Postoperative neurocognitive dysfunction (PND) is a prevalent and debilitating complication in elderly surgical patients, characterized by persistent cognitive decline that negatively affects recovery and quality of life. As the aging population grows, the rising number of elderly surgical patients has made PND an urgent clinical challenge. Despite increasing research efforts, the pathophysiological mechanisms underlying PND remain inadequately characterized, underscoring the need for a more integrated framework to guide targeted interventions. To better understand the molecular mechanisms and therapeutic targets of PND, this narrative review synthesized evidence from peer-reviewed studies, identified through comprehensive searches of PubMed, Embase, Cochrane Library, and Web of Science. Key findings highlight neuroinflammation, oxidative stress, mitochondrial dysfunction, neurotransmitter imbalances, microvascular changes, and white matter lesions as central to PND pathophysiology, with particular parallels to encephalocele- and sepsis-associated cognitive impairments. Among these, neuroinflammation, mediated by pathways such as the NLRP3 inflammasome and blood–brain barrier disruption, emerges as a pivotal driver, triggering cascades that exacerbate neuronal injury. Oxidative stress and mitochondrial dysfunction synergistically amplify these effects, while neurotransmitter imbalances and microvascular alterations, including white matter lesions, contribute to synaptic dysfunction and cognitive decline. Anesthetic agents modulate these interconnected pathways, exhibiting both protective and detrimental effects. Propofol and dexmedetomidine demonstrate neuroprotective properties by suppressing neuroinflammation and microglial activation, whereas inhalational anesthetics like sevoflurane intensify oxidative stress and inflammatory responses. Ketamine, with its anti-inflammatory potential, offers promise but requires further evaluation to determine its long-term safety and efficacy. By bridging molecular insights with clinical practice, this review highlights the critical role of personalized anesthetic strategies in mitigating PND and improving cognitive recovery in elderly surgical patients. It aims to inform future research and clinical decision-making to address this multifaceted challenge.

## 1. Introduction

Postoperative neurocognitive dysfunction (PND) is a prevalent and significant complication in elderly surgical patients, characterized by cognitive decline that impairs recovery and negatively affects quality of life [1,2,3]. With the global aging population and an increasing number of elderly patients undergoing surgeries [4,5], understanding the mechanisms underlying PND has become an urgent clinical challenge. Despite extensive research into the causes of PND, its exact pathophysiology remains poorly understood, and effective prevention and treatment strategies are still lacking.

Several reviews, such as those by Liu et al. [6] and Zhao et al. [7], have provided valuable insights into PND by exploring its clinical risk factors and specific molecular mechanisms. Building on their findings, this review integrates these insights into a comprehensive framework that links pathophysiology to therapeutic strategies. It adopts a uniquely systematic perspective by synthesizing key processes implicated in PND, including neuroinflammation, neurodegeneration, oxidative stress, mitochondrial dysfunction, neurotransmitter imbalances, microvascular changes, and white matter lesions. In addition, this review critically examines the dual roles of anesthetic agents—such as propofol, dexmedetomidine (DEX), and inhalational anesthetics—in modulating these mechanisms, highlighting both their neuroprotective potential and neurotoxic risks within the context of PND. Furthermore, emerging parallels between PND and related conditions, including encephalocele and sepsis-associated encephalopathy (SAE), provide valuable insights into overlapping and distinct mechanisms that may inform broader therapeutic strategies.

This narrative review bridges critical gaps in the literature by combining molecular insights with clinical practice, offering a holistic framework for understanding the interconnected pathways driving PND. We hypothesize that this integrative perspective will inform more effective, targeted therapeutic strategies to mitigate PND and improve cognitive recovery in elderly surgical patients. Given the heterogeneity in study designs, patient populations, and mechanistic focuses across the current literature, this review adopts a narrative approach to synthesize diverse findings, providing a comprehensive understanding of PND and its implications for perioperative care.

## 2. Materials and Methods

This narrative review aimed to synthesize insights into the molecular and cellular mechanisms of postoperative neurocognitive dysfunction (PND) and assess the effects of anesthetic agents on these mechanisms. The review follows established guidelines for narrative reviews, ensuring a systematic and comprehensive approach to synthesizing the existing literature. A comprehensive search was conducted using PubMed/Medline, Embase, Cochrane Library, and Web of Science databases to retrieve relevant literature on postoperative neurocognitive dysfunction (PND) published between January 2000 and December 2024. The search was performed using a combination of Medical Subject Headings (MeSH) terms and keywords related to the topic. The MeSH terms used in the search include the following: #1 = Cognitive Dysfunction, #2 = Postoperative Complications, #3 = Neuroinflammation, #4 = Oxidative Stress, #5 = Mitochondria, #6 = Oxidative Stress, #7 = Microcirculation, #8 = Blood–Brain Barrier, #9 = White Matter, #10 = Encephalocele, #11 = Sepsis-associated Encephalopathy (SAE), #12 = Capillaries, #13 = Anesthetics, #14 = Propofol, #15 = Dexmedetomidine, #16 = Sevoflurane, and #17 = Ketamine. This review used additional keywords such as “Postoperative Cognitive Dysfunction”, “Postoperative Neurocognitive Disorder”, “Neurodegeneration”, “Neurotransmitter Imbalance”, “Mitochondrial Dysfunction”, “Microvascular Changes”, and “White Matter Lesions”, to capture relevant studies beyond exact MeSH terms. A manual search was also performed to identify additional works not captured by the initial strategy. All works published in English between 2000 and 2024 were considered. Boolean operators were used to refine the search strategy, ensuring relevant and precise results.

Inclusion and Exclusion Criteria: Studies were included if they met the following criteria: (1) Were original articles published in peer-reviewed journals, including experimental studies, clinical trials, or systematic reviews exploring the molecular and cellular mechanisms of PND. (2) Focused on anesthetic agents’ effects on PND mechanisms, specifically in neuroinflammation, oxidative stress, mitochondrial dysfunction, neurodegeneration, neurotransmitter imbalances, microvascular changes, white matter lesions, encephalocele, or sepsis-associated encephalopathy (SAE). (3) Included studies on human subjects or animal models, particularly elderly surgical patients. (4) Were published in English and provided adequate methodological details. Studies were excluded if they met the following criteria: (1) Focused on unrelated topics or did not address molecular mechanisms or anesthetic effects in PND. (2) Were non-original articles, such as editorials, case reports, or letters to the editor. (3) Were not peer-reviewed, such as conference abstracts or unpublished manuscripts. Data Extraction and Synthesis: Data were extracted on key mechanisms involved in PND, including neuroinflammation, oxidative stress, mitochondrial dysfunction, neurotransmitter imbalances, microvascular changes, and white matter lesions, as well as conditions like encephalocele and sepsis-associated encephalopathy (SAE). The role of anesthetic agents in these processes was also analyzed. Findings from animal and clinical studies on the effects of anesthetics on cognitive function and recovery in elderly surgical patients were synthesized narratively. No statistical analyses were conducted, as this review employs a qualitative synthesis approach to highlight the mechanisms of PND and the clinical implications of anesthetic management.

## 3. Results

### 3.1. Potential Mechanisms of PND

**Figure 1:**
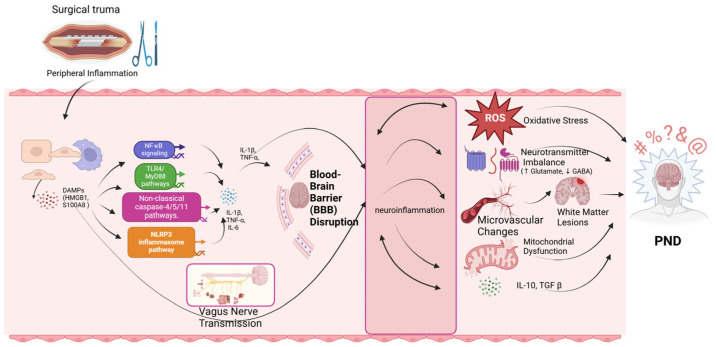
Surgical trauma serves as the starting point of the process, causing tissue damage and releasing damage-associated molecular patterns (DAMPs). These molecules activate the peripheral immune system, leading to peripheral inflammation, marked by the release of inflammatory mediators, including IL-6 and TNF-α. Peripheral inflammation affects the central nervous system (CNS) through several pathways: Neuroinflammation: Peripheral inflammatory signals are transmitted via the vagus nerve or directly through the weakened BBB, activating microglia and astrocytes in the brain, further amplifying the inflammatory response. Neuroinflammation triggers downstream effects. Oxidative Stress: Elevated production of reactive oxygen species (ROS) damages lipids, proteins, and DNA, exacerbating neuronal injury. Blood–Brain Barrier (BBB) Disruption: Inflammatory cytokines and immune cells penetrate the brain as the BBB integrity is compromised. Mitochondrial Dysfunction: Impaired mitochondrial function disrupts energy metabolism and increases ROS production, creating a vicious cycle of oxidative stress and cellular damage. Neurotransmitter Imbalance: Neuroinflammation and oxidative stress alter neurotransmitter dynamics, including excessive release of glutamate and reduced GABA levels, leading to excitotoxicity and neuronal hyperexcitability. Microvascular Dysfunction: Inflammatory mediators impair microvascular integrity, leading to hypoxia and reduced nutrient supply to the brain. Microvascular dysfunction further contributes to the development of white matter lesions, where damage to myelinated axons disrupts the connectivity between brain regions. These lesions are particularly common in the aging brain, where pre-existing cerebrovascular risk factors such as hypertension and diabetes exacerbate the damage. The combined effects of oxidative stress, mitochondrial dysfunction, neurotransmitter imbalances, and white matter lesions ultimately lead to cognitive dysfunction, manifested as impairments in memory, attention, and overall cognitive function.

Postoperative neurocognitive dysfunction (PND) is a multifaceted pathological condition, and its precise mechanisms remain incompletely understood. Current research suggests that the occurrence of PND primarily involves several mechanisms, including neuroinflammation, neurodegeneration, oxidative stress and mitochondrial dysfunction, neurotransmitter imbalance, microvascular function changes, and white matter lesions. The individual effects of these mechanisms or their interactions may lead to postoperative neurocognitive dysfunction. This review comprehensively integrates and summarizes the latest research on the mechanisms underlying postoperative neurocognitive dysfunction (PND) (Figure 1).

### 3.2. Neuroinflammation

#### 3.2.1. Surgically Induced Neuroinflammation

Surgery frequently initiates an acute inflammatory reaction, often accompanied by symptoms such as fever and lethargy [8]. The mechanical injury caused by surgery is an unavoidable outcome, which activates the innate immune system, resulting in an inflammatory reaction. This reaction is a fundamental aspect of the body’s natural defense against trauma and plays a significant role in the clinical symptoms observed in many postoperative patients [9]. Surgery itself can induce tissue injury and inflammatory processes. Surgical trauma releases cellular molecules collectively referred to as damage-associated molecular patterns (DAMPs), which further activate the innate immune system [10,11].

The release of DAMPs, including CRP, S100A8, and HMGB1, as a result of sterile trauma caused by surgery, stimulates immune cells such as monocytes and macrophages. These cells, in turn, activate inflammatory signaling pathways like NF-κB, HMGB1/TLR pathways, and NLRP3/caspase-1 pathways, and the non-classical caspase-4/5/11 pathways. Various inflammatory mediators, including cytokines and chemokines, are released through interactions with pattern recognition receptors (PRRs) such as TLR2 and TLR4 [11,12,13,14,15,16,17]. Additionally, animal studies have shown that these processes can induce the accumulation of CCR2-expressing macrophages in the hippocampus, followed by upregulation of CCL2 in activated astrocytes and CCR2 in activated microglia, which contribute to the development of PND [12,18].

The blood–brain barrier (BBB) plays a crucial role in restricting the entry of inflammatory agents into the central nervous system (CNS). However, peripheral inflammation can disrupt BBB integrity, allowing blood-borne cytokines to infiltrate the brain and initiate neuroinflammation [19,20,21]. Peripheral immune signals may also reach the brain via sensory neurons in the brainstem, contributing to neuroinflammation [22,23]. The extent of the postoperative systemic inflammatory response is related to the severity of surgical trauma. Therefore, excessive peripheral inflammation can significantly affect the brain [24,25,26]. Even minor surgeries can induce neuroinflammation in the elderly [27]. When the BBB remains intact, pro-inflammatory cytokines can infiltrate the central nervous system through periventricular areas or via active transport mechanisms. IL-1 can activate matching receptors on endothelial cells of the BBB, which then triggers the secretion of pro-inflammatory agents (such as TNF, IL-6, IL-1, and PGE2) into the brain parenchyma [28,29]. This process compromises BBB integrity, allowing peripheral inflammatory cytokines and immune cells to enter the central nervous system, ultimately resulting in neuroinflammation [30,31].

Surgical trauma triggers a systemic inflammatory response, which enhances the synthesis and release of pro-inflammatory mediators. These mediators can accumulate in the central nervous system, particularly in areas with high immune activity or existing damage, such as the hippocampus [25,32,33]. The amplification of neuroinflammation following surgery is partly mediated by the permeable BBB, along with contributions from vagal afferent nerves and additional factors. The vagus nerve transmits inflammatory signals from the gut, liver, lungs, and other organs to the brain [34,35,36,37,38]. Overproduction of neurotransmitter glutamate by vagal nerve endings activates mast cells through NR2B receptors [35]. As key neuroimmune cells, mast cells mediate the interplay between the nervous and immune systems [39]. Mast cells in the brain play a crucial role in neuroinflammation and PND, acting as primary responders to brain injury and environmental sensors [37]. By releasing inflammatory and toxic mediators, these cells stimulate nearby glial cells and neurons [35,36,37]. TNF-α, by binding to the TNFR death receptor and triggering a series of responses, leads to cell death and further activation of microglia, promoting the formation of a chronic neuroinflammatory environment [40]. At the early stage of trauma, surgical procedures may induce C-reactive protein (CRP) release, potentially activating complement component 3 (C3) and initiating coagulation and fibrinolysis processes [41,42]. The complement system and coagulation cascade are activated by surgical trauma, thereby expediting the inflammatory response [43]. Post-surgery, HMGB1 enters the bloodstream swiftly, initiating the NF-κB signaling pathway through its interaction with RAGE [44], TLR4 [32], and TLR2 [17]. This leads to the release of cytokines like TNF-α, IL-1β, IL-18, and IL-6, which are key pro-inflammatory mediators. S100A8 can promote the activation of the TLR4/MyD88 signaling pathway in macrophages [45], thereby promoting a positive feedback loop of HMGB1 and S100A8 production, sustaining and intensifying the systemic inflammatory response [15,46,47]. Consequently, peripheral sterile inflammation resulting from surgical trauma triggers CNS neuroinflammation through these pathways and mechanisms, contributing to the onset of PND.

The mechanisms outlined above have been thoroughly investigated in animal models and are relatively well established; however, clinical research remains in its early stages. Nonetheless, several clinical studies have validated the association between surgery-induced inflammatory responses and postoperative neurocognitive dysfunction (PND). The findings indicate that elevated postoperative inflammatory markers are strongly associated with the occurrence of PND. Inflammatory markers in the postoperative serum, such as interleukin-1β (IL-1β), S100 calcium-binding protein β (S-100β), serum amyloid A (SAA), and high-mobility group box 1 protein (HMGB1), are significantly elevated in PND patients [48,49]. These marker levels are significantly higher in PND patients at various postoperative time points (e.g., 1 h, 6 h, 24 h, day 1, and day 3 postoperatively) compared to those without PND, suggesting that these inflammatory markers may serve as important predictors of PND.

However, some clinical studies have pointed out that although HMGB1 levels increase on the first postoperative day, this increase is not significantly correlated with the severity of delirium [50]. The authors explain that although HMGB1 levels increase on the first postoperative day, failure to capture the true peak of HMGB1 may prevent an accurate assessment of its relationship with delirium. This explains why an increase in HMGB1 levels was observed without a significant correlation with delirium severity. Therefore, the clinical relevance of the surgically induced inflammatory response mechanism requires further confirmation.

In clinical studies, unavoidable anesthesia factors may also influence the increase in these inflammatory markers. To eliminate this influence, studies have found that even in elderly patients who did not undergo surgery, the use of sevoflurane alone for general anesthesia did not cause an acute increase in biomarkers, indicating that sevoflurane does not induce significant inflammatory states or neural damage [51]. Other studies have indicated that administering propofol or sevoflurane alone for general anesthesia may not have a significant effect on cognitive recovery in healthy adults across all age groups. These results suggest that the surgical process itself, rather than anesthetic factors, may be the primary cause of elevated neuroinjury biomarkers and postoperative neurocognitive dysfunction. This offers valuable biochemical insights into the effects of general anesthesia on healthy adults, emphasizing the distinct roles played by surgery and anesthesia [52].

#### 3.2.2. The Impact of Anesthetic Agents on Neuroinflammation

##### Opioids and Neuroinflammation

Opioids can influence the central nervous system’s susceptibility to infections. In animal models, opioids engage with multiple receptors on microglia, including TLR4/MD2, P2XR, and μ-opioid receptor (μ-OR). This interaction triggers microglial activation, leading to the release of nitric oxide (NO) and pro-inflammatory cytokines, which contribute to neurotoxic effects [53,54,55]. Additionally, opioids can amplify the TLR4 signaling pathway via microglial activation, further stimulating the production of inflammatory factors such as TNF-α and IL-6. These substances are capable of crossing the blood–brain barrier, intensifying brain inflammation and impairing cognitive function [56,57].

Clinical studies have indicated that in patients undergoing fast-track total hip arthroplasty (THA) and total knee arthroplasty (TKA), the occurrence of PND may be linked to postoperative opioid use, which aligns with the current trend towards opioid-sparing analgesia [58]. Another study highlighted that patients using long-term opioid analgesics exhibited significantly reduced attention performance (*p* < 0.05) and diminished beliefs in their ability to manage pain effectively (*p* < 0.05) compared to those not taking opioids. However, despite long-term opioid use, there was no difference in pain severity or pain scores, memory, or average plasma cytokine concentrations [59]. Nonetheless, other studies indicate that in patients with opioid use disorder, an analysis of memory ability shows a negative correlation with TNF-α and IL-6 levels [60]. Further clinical investigations are required to clarify the specific mechanisms that connect opioid use with postoperative neurocognitive dysfunction.

##### Propofol and Neuroinflammation

Propofol is a widely used intravenous anesthetic known for its complex neuroprotective and anti-inflammatory properties, although it may lead to cognitive impairment under certain conditions.

Neuroprotective and Anti-inflammatory Properties of Propofol: Propofol may provide neuroprotection by influencing the activity of brain-derived neurotrophic factor (BDNF) and its receptor TrkB [61]. It has been shown to decrease the production of inflammatory cytokines by suppressing the NF-κB signaling pathway [62] and to mitigate microglial activation by modulating the MAPK ERK1/2 pathway, thereby reducing the release of pro-inflammatory cytokines. Propofol may also activate the PI3k/Akt pathway, promoting microglial survival and inhibiting GSK-3β activity through Akt phosphorylation, thereby reducing the inflammatory response [63,64], thus exerting anti-inflammatory effects.

Propofol-mediated Neuroinflammation: Although propofol can exert anti-inflammatory and neuroprotective effects through the pathways mentioned above, it may also trigger neuroinflammation and stimulate microglial activation, with the extent of these effects being age-dependent [65,66]. Particularly in the developing brain, where synaptogenesis is high, propofol may disrupt synaptic development, increase neuronal apoptosis, and potentially lead to developmental neurotoxicity, impairing cognitive function [61,65,66,67]. These effects are associated with changes in key proteins such as ApoE and Tau, which are involved in neuroinflammatory responses [68]. Prolonged exposure to or high concentrations of propofol can trigger microglial activation, which may be associated with the suppression of BDNF expression in the hippocampus. This, in turn, disrupts the BDNF/TrKB signaling pathway and its downstream PI3K/Akt activation, ultimately promoting apoptosis [61]. Research has shown that repeated propofol administration might lead to long-term cognitive deficits, heightened pro-inflammatory responses, and increased activation of NF-κB and NLRP3 inflammasomes in the brain [65,69], leading to cognitive dysfunction. These mechanisms have been explored primarily in animal experiments.

Previously, numerous clinical studies have investigated the effects of propofol anesthesia compared to inhalation anesthesia on PND in elderly patients [70,71,72,73,74]. However, the results remain controversial. Several studies and meta-analyses have reported that propofol can reduce the incidence of PND and improve cognitive function in postoperative patients, with potential anti-inflammatory effects [74,75]. However, other clinical studies and meta-analyses have found no difference in the incidence of PND between the propofol anesthesia group and the sevoflurane inhalation group, or have failed to reach a definitive conclusion proving any difference between the two [76,77,78]. Additionally, some studies examining the effects of propofol versus sevoflurane anesthesia on cognitive function in lung surgery patients suggest that propofol may have a more negative impact on cognitive function than sevoflurane [79,80].

Therefore, whether propofol has a protective or harmful effect on cognitive function remains controversial. Clinical study results only show the impact of propofol anesthesia combined with surgery on cognitive function, not the isolated effects of anesthesia or surgery. Some studies propose that postoperative inflammatory responses, influenced by patient and surgical factors, may play a more significant role in cognitive dysfunction compared to anesthetic factors [81]. Therefore, based on both basic and clinical research, it has been found that factors such as inflammatory response, age, propofol dosage, anesthesia depth, propofol anesthesia duration, and intervals may all play a role in regulating propofol’s effects on cognitive function. However, the exact mechanisms through which propofol influences cognitive function as well as the interactions among various factors still require further research [82].

##### Ketamine and Neuroinflammation

Ketamine is an NMDA receptor antagonist commonly used for short-term anesthesia and analgesia. Animal studies have shown that the anti-inflammatory properties of ketamine are mainly reflected in the inhibition of microglial activation [83,84]. This process may occur via the MAPK ERK1/2 pathway [84]. By modulating the P38MAPK pathway, ketamine can reduce microglial activation in the hippocampus and decrease the production of TNF-α, NO, and IL-1β [85,86]. Additionally, ketamine may exert neuroprotective effects by blocking NMDA receptors, promoting synaptic release of BDNF, activating the TrkB pathway, and inhibiting NF-κB translocation [87]. Both esketamine and ketamine have been shown to alleviate inflammation through various pathways, primarily by decreasing the levels of pro-inflammatory cytokines, such as TNF-α and IL-6 [88,89,90,91].

Clinical research has found that in patients undergoing cardiac surgery associated with ketamine (intravenous injection of 0.5 mg/kg during anesthesia induction), the incidences of postoperative neurocognitive dysfunction and inflammatory markers were significantly lower compared to the control group [92]. Another study found that sub-anesthetic doses of esketamine might reduce the incidence of delayed neurocognitive recovery in elderly patients undergoing gastrointestinal surgery and improve early postoperative cognitive function, potentially due to its anti-neuroinflammatory effects [93].

##### Dexmedetomidine and Neuroinflammation

Dexmedetomidine, an α2-adrenergic agonist, exhibits significant anti-inflammatory properties and has shown notable protective effects in preventing and ameliorating postoperative neurocognitive dysfunction (PND). Based on animal studies, dexmedetomidine can reduce the conversion of microglia to the M1 phenotype, stimulate astrocyte production of BDNF, and decrease NLRP3 activation [94,95]. Dexmedetomidine also inhibits the activation of the NLRP3 inflammasome and reduces the expression of inflammatory cytokines such as IL-6 and TNF-α, thereby alleviating postoperative neuroinflammation and oxidative stress. Studies have demonstrated that dexmedetomidine exerts neuroprotective effects against postoperative cognitive decline via the NLRP3 inflammasome signaling pathway [96]. Moreover, dexmedetomidine can regulate the P38MAPK pathway, reducing microglial activation and the production of inflammatory mediators such as NO, TNF-α, and IL-1β [97].

Clinical studies have explored the effects of dexmedetomidine on postoperative cognitive dysfunction in elderly patients undergoing orthopedic surgery through randomized controlled trials [98]. Dexmedetomidine was shown to protect cognitive function by reducing postoperative inflammation and oxidative stress, protecting the BBB, and lowering the risk of neuronal injury, leading to improved postoperative cognitive function, including enhanced memory and learning ability and reduced postoperative depression symptoms [98]. Clinical trials have validated the efficacy and safety of dexmedetomidine in improving postoperative cognitive dysfunction in elderly orthopedic surgery patients. These mechanisms suggest that dexmedetomidine can effectively prevent and mitigate postoperative cognitive dysfunction, provide neuroprotection, and improve overall cognitive function and quality of life in postoperative patients. However, a few studies have noted that the improvement of postoperative neurocognitive function in elderly patients undergoing total knee arthroplasty with dexmedetomidine is unrelated to its regulation of peripheral inflammation [99]. The clinical research mechanisms of dexmedetomidine in neuroinflammation require further exploration.

##### Inhalational Anesthetics and Neuroinflammation

Basic research has found that inhalational anesthetics such as isoflurane and sevoflurane can activate microglia in the central nervous system. Activated microglia release pro-inflammatory cytokines like IL-1β, TNF-α, and IL-6, leading to neuroinflammation. Neuroinflammation can impair neuronal function and synaptic plasticity, resulting in cognitive decline [62,100]. Inhalational anesthetics can also induce oxidative stress, increase reactive oxygen species (ROS) production, further stimulate microglial activation, and release inflammatory cytokines, exacerbating neuroinflammation. These oxidative stress responses damage neurons and increase cell death, further impairing cognitive function [101,102]. Inhalational anesthetics may also damage the BBB, increasing its permeability, allowing peripheral inflammatory factors to enter the central nervous system, and promoting the development of neuroinflammation. The disruption of the BBB allows peripheral immune cells and inflammatory cytokines to more easily enter the brain, triggering or exacerbating neuroinflammation [32]. These mechanisms collectively trigger or exacerbate postoperative neurocognitive dysfunction (PND).

Numerous clinical studies have confirmed that sevoflurane leads to cognitive dysfunction, but a few studies have pointed out that the dose of sevoflurane is unrelated to the severity or incidence of delirium. The occurrence of delirium may be due to other biological mechanisms, such as inflammation and neuronal damage, rather than the dose of sevoflurane [81]. However, as these studies were conducted under the combined influence of surgery and other factors, the single effect of anesthetic agents still requires further clinical research.

### 3.3. Neurodegeneration

Basic research has shown that neuroinflammation caused by surgery and anesthetic factors can result in neuronal damage and apoptosis. The loss of neurons and a reduction in synapses negatively impact cognitive function in the brain [32,37]. The neurodegenerative process also involves neurotransmitter imbalances, mitochondrial dysfunction, and oxidative stress [103,104]. Neurotransmitter imbalances, such as excessive release of glutamate and decreased levels of GABA, lead to neuronal hyperexcitability and excitotoxicity [32,102]. Mitochondrial dysfunction and oxidative stress also play crucial roles in neurodegeneration. Mitochondrial dysfunction results in insufficient energy supply and increased ROS production, triggering oxidative stress and cellular damage [105,106], leading to cognitive dysfunction.

Clinically, studies on neurodegeneration and postoperative neurocognitive dysfunction (PND) mainly rely on biomarkers of neuronal degeneration and indirect evidence. For example, in patients undergoing elective laparoscopic cholecystectomy under general anesthesia, it was found that the occurrence of PND was associated with elevated levels of serum neuronal degeneration markers such as S100B [107]. Therefore, the clinical mechanisms of neurodegeneration in PND require further exploration.

### 3.4. Oxidative Stress and Mitochondrial Dysfunction

Basic research has shown that the stress response and systemic inflammatory response induced by surgery and anesthesia can increase the production of reactive oxygen species (ROS), leading to damage to lipids, proteins, and DNA in neurons [108]. This results in cellular dysfunction or death, exerting neurotoxic effects [108,109,110]. Sustained oxidative stress may lead to long-term neuronal damage and inflammation [111,112,113,114]. In the brain, oxidative stress is particularly harmful to neurons, potentially directly affecting their survival and function [115]. Mitochondria are the primary energy suppliers and major sources of ROS production. Mitochondrial dysfunction leads to insufficient energy supply, further increasing ROS production, creating a vicious cycle [116,117]. Inhalational anesthetics can cause mitochondrial dysfunction, increase the production of ROS, and thus induce oxidative stress responses. These responses damage the energy metabolism of neurons, leading to apoptosis and cognitive decline [118]. Oxidative stress can also disrupt the integrity of the blood–brain barrier, allowing pro-inflammatory mediators and immune cells to enter the central nervous system, exacerbating neuroinflammation [119].

Clinically, patients undergoing laparoscopic gastrointestinal tumor surgery were included in a study. A UPLC/MS-based serum metabolomics method was established to study the metabolic changes in PND patients. Forty differential metabolites were identified. IPA analysis of the interaction network of these differentially expressed metabolites in PND suggested that mitochondrial dysfunction might be one of the causes of PND [120]. Additionally, another article suggested that dexmedetomidine might alleviate neuronal damage possibly caused by mitochondrial membrane oxidative stress, reducing the damage to mitochondrial enzyme systems, and ultimately improving postoperative neurocognitive dysfunction [121].

### 3.5. Neurotransmitter Imbalance

Basic research suggests that neurotransmitter imbalance is another important mechanism in the occurrence of PND, mainly involving changes in the balance between glutamate and GABA. During surgery and anesthesia, excessive release of glutamate and decreased levels of GABA can lead to neuronal hyperexcitability and neurotoxicity, directly affecting cognitive function [122]. For example, increased levels of glutamate can lead to excitotoxicity, causing neuronal damage and death, while decreased GABA levels reduce the inhibitory effect on this excitotoxicity, further exacerbating neuronal damage and cognitive decline [123]. These findings suggest that modulating the balance between glutamate and GABA might be a potential strategy for preventing and treating PND. During surgery and anesthesia, excessive release of glutamate leads to neuronal hyperexcitability, and prolonged high levels of glutamate can damage neurons, a phenomenon known as excitotoxicity, affecting cognitive function, particularly memory and attention [124].

Clinical evidence confirms that hepatic encephalopathy is based on the theory of increased GABAergic tone, leading to cognitive dysfunction. In patients with liver cirrhosis and certain cases of hepatic encephalopathy, the administration of flumazenil (a highly selective benzodiazepine antagonist for GABA receptors) has been shown to enhance EEG activity, reverse coma, and alleviate hypoactive delirium symptoms. These effects confirm the involvement of neurotransmitter imbalances in cognitive dysfunction [125]. Meanwhile, increasing clinical evidence shows that antipsychotic drugs are effective for delirium [126,127,128,129,130,131]. A recent meta-analysis also suggested that antipsychotics are among the few drugs proven to be effective in preventing delirium [132]. Additionally, some studies indicated that low-dose risperidone could treat postoperative delirium. Although risperidone’s primary mechanism does not directly involve the glutamate and GABA systems, it may indirectly affect these systems through its influence on the dopamine and serotonin systems, impacting central nervous system activity and mental status [133]. This further confirms the significant role of neurotransmitters in postoperative cognitive dysfunction.

### 3.6. Microvascular Function Changes

Surgery-induced systemic inflammatory responses can compromise the integrity of the blood–brain barrier (BBB), allowing pro-inflammatory cytokines and immune cells to enter the central nervous system, leading to neuroinflammation. Basic research indicates that the systemic inflammatory response generated during surgery and anesthesia can increase BBB permeability, allowing inflammatory mediators and immune cells to infiltrate the brain, activate glial cells, and further trigger and exacerbate neuroinflammation [134]. Additionally, reduced microvascular blood flow can lead to hypoxia and malnutrition in local brain tissue, affecting the health and function of neurons. Reduced blood flow and hypoxic conditions can trigger a series of neuropathological changes, including neuronal damage and death [134]. Metabolic syndrome and its components (such as hypertension and diabetes) increase the risk of cerebrovascular diseases, further affecting cerebral blood flow and cognitive function. Hypertension and diabetes, among other metabolic diseases, can worsen vascular wall damage, leading to cerebral hypoperfusion and increasing the risk of cognitive dysfunction [135].

Clinical studies have examined patients undergoing cardiac surgery (coronary artery bypass grafting (CABG), heart valve surgery, or CABG plus valve surgery) and non-cardiac surgery, including 10 cardiac surgery patients and 8 non-cardiac surgery patients. Pre- and postoperative magnetic resonance imaging (MRI) was used to assess BBB permeability using a delayed contrast extravasation subtraction method. The study found that nearly half of the surgical patients experienced increased BBB permeability postoperatively, with a potential link to lower cognitive performance [136]. Abrahamov reached similar conclusions in another study [137].

Additionally, clinical studies employing the cerebrospinal fluid-to-plasma albumin ratio (CPAR) to evaluate BBB dysfunction revealed that increased postoperative BBB permeability was associated with delirium incidence [138]. Another study by Jennifer Taylor [139] further demonstrated that elevated perioperative CPAR and S100B levels correlated with delirium severity, while a reduction in S100B levels was predictive of symptom recovery (*p* < 0.001). Linear regression analysis indicated that plasma S100B changes were independently connected to surgical risk, cardiovascular procedures, blood loss, and hypotension. Blood loss was also correlated with CPAR, S100B, cerebrospinal fluid lactate, and peak delirium severity, aligning with prior research findings. This finding also helps to elucidate the mechanism by which intraoperative hypotension leads to postoperative cognitive dysfunction observed in clinical settings. Additionally, clinical studies suggest that dexmedetomidine may lower the incidence of postoperative delirium in elderly patients with mild cognitive impairment by reducing blood–brain barrier permeability and neuroinflammation [140].

### 3.7. White Matter Lesions

White matter primarily consists of myelinated axons, along with oligodendrocytes, astrocytes, microglia, blood vessels, and other components [141,142]. The myelinated axons in white matter are covered by a fatty substance called myelin, giving white matter its characteristic color. Myelin not only protects axons but also speeds up the transmission of nerve impulses [143,144,145,146]. Myelinated axons, grouped into large bundles within white matter, facilitate communication between various brain regions and between the brain and spinal cord.

Maintaining the structural and functional integrity of white matter requires the coordinated action of the neurovascular unit and the glymphatic system to provide sufficient energy, regulate cellular electrolyte and nutrient homeostasis, and clear metabolic waste [147]. The neurovascular unit and the glymphatic system are closely linked in maintaining neuronal homeostasis, providing oxygen, energy, water, and nutrients [147]. With aging, due to cumulative damage to brain structures and the loss of neurovascular–glymphatic function, compensatory mechanisms gradually fail, increasing the vulnerability of white matter to these damages [148].

Hypertension, diabetes, hyperlipidemia, and hyperhomocysteinemia collectively contribute to white matter lesions (WMLs) through abnormalities in the neurovascular unit (such as disruption of the blood–brain barrier, reduced cerebral blood flow, and microvascular disease) and the glymphatic system (such as the accumulation of metabolic waste and dysfunction of AQP4 water channels) [147,149,150]. Dysfunction in certain components of the glymphatic system, such as AQP4 water channels, can directly affect oligodendrocytes, leading to the development and progression of WMLs [147], which in turn may produce direct delirium effects [147]. Recent studies have shown that preoperative WMLs are a significant risk factor for severe postoperative delirium and PND in humans. Interestingly, cerebrovascular risk factors such as age, hypertension, diabetes, pre-existing brain, heart, or vascular diseases, hyperlipidemia, hyperhomocysteinemia, and elevated high-sensitivity C-reactive protein are common risk factors for both WMLs and PND [151,152,153]. Poor sleep quality is also associated with cerebrovascular dysregulation and WMLs. Studies have found that periodic impairment of cerebral blood flow during sleep is associated with WMLs, and research consistently shows that poor sleep quality is related to cerebrovascular dysregulation and WMLs. These mechanisms also explain the clinical observations of hypertension, diabetes, pre-existing brain, heart, or vascular diseases, hyperlipidemia, and poor sleep quality leading to postoperative neurocognitive dysfunction [154,155].

Table 1 provides a summary of the key mechanisms, anesthetic effects, and potential therapeutic strategies for PND.

### 3.8. Exploration of Distinct Yet Partially Overlapping Mechanisms in PND, Encephalocele, and SAE

Emerging parallels between PND, encephalocele, and sepsis-associated encephalopathy (SAE) provide valuable insights into their distinct yet partially overlapping pathophysiology. Encephalocele, the protrusion of brain tissue and/or meninges through cranial defects, frequently requires surgical correction. Postoperative complications, such as infection, cerebrospinal fluid (CSF) leakage, elevated intracranial pressure, and developmental delays, can contribute to central nervous system (CNS) vulnerability and impact neurocognitive outcomes. In particular, postoperative infections and CSF leakage may contribute to neuroinflammation by amplifying systemic immune responses, which can indirectly influence CNS function [156]. Further research is needed to clarify how these complications might affect neurocognitive outcomes, particularly in relation to PND [157] and other neuroinflammatory conditions like SAE.

Sepsis-associated encephalopathy (SAE) arises from systemic inflammation during sepsis, which triggers the release of pro-inflammatory mediators and may impair blood–brain barrier (BBB) integrity. This impairment contributes to neuroinflammatory responses within the central nervous system (CNS), leading to neuronal dysfunction. Additionally, inflammation induces oxidative stress, which exacerbates mitochondrial dysfunction. Together, these interconnected processes—neuroinflammation, oxidative stress, and mitochondrial damage—play critical roles in the development of cognitive impairment in SAE [158]. A recent study by Tian et al. [159] demonstrated that DEX improved cognitive function in a murine model of SAE induced by cecal ligation and puncture (CLP). Specifically, DEX treatment improved cognitive function, reduced BBB disruption, and attenuated hippocampal inflammation and neuronal apoptosis. These findings highlight DEX’s potential as a therapeutic agent for mitigating sepsis-related neurocognitive impairments by targeting key pathological processes, including BBB dysfunction and neuroinflammation.

These distinct yet partially overlapping mechanisms highlight the interconnected processes underlying PND, encephalocele, and SAE. Insights from these related conditions could inform therapeutic strategies to mitigate neurocognitive dysfunction by addressing shared pathways, such as neuroinflammation, while considering condition-specific factors.

## 4. Discussion

This narrative review highlights the multifactorial nature of PND and identifies key mechanisms, including neuroinflammation, neurodegeneration, oxidative stress, mitochondrial dysfunction, and microvascular changes, and white matter lesions as central contributors to its pathophysiology. It emphasizes the dual roles of anesthetic agents—such as dexmedetomidine, propofol, and inhalational anesthetics—which can exhibit both neuroprotective and neurotoxic effects depending on dosage, timing, and context of use. Additionally, this review integrates insights from related conditions, such as encephalocele and sepsis-associated encephalopathy (SAE), to provide a broader understanding of the distinct yet partially overlapping mechanisms contributing to neurocognitive dysfunction. These insights reinforce the importance of targeted therapeutic strategies to mitigate PND and improve recovery.

A major strength of this review is its comprehensive approach, which synthesizes molecular, cellular, and clinical perspectives to create a unified framework for understanding PND. By integrating findings across different levels of research, this review bridges gaps between basic mechanisms and clinical practice. Furthermore, the inclusion of encephalocele and SAE offers a comparative perspective, highlighting how localized encephalocele and systemic SAE factors can converge on shared pathways, such as neuroinflammation, while retaining distinct pathophysiological features.

It is important to acknowledge that this review is qualitative in nature and does not possess the methodological rigor of a systematic review, which would involve a structured approach and quantitative synthesis, such as meta-analyses. We deliberately opted for a narrative review format, as it allows for a nuanced and flexible exploration of the complex and multifactorial mechanisms underlying PND. Given the heterogeneity of the current literature, future systematic reviews focusing on specific aspects—such as the long-term effects of individual anesthetic agents or neuroinflammatory pathways—could provide more robust statistical insights. Despite this limitation, we believe that the narrative approach adds value by offering a broader perspective on the interconnected mechanisms of PND and their clinical implications. Additionally, the inclusion of encephalocele and SAE within this framework highlights key research gaps, including the need for targeted studies on the roles of systemic inflammation and other postoperative complications in PND development. As PND is inherently multifactorial, the narrative format provided the flexibility needed to discuss the interplay between these mechanisms and their broader clinical relevance. Future systematic reviews could focus on more targeted topics, such as the effects of specific anesthetic agents or a focused quantitative analysis of neuroinflammation in PND.

The findings of this review have significant clinical implications, especially in improving the management of PND in elderly surgical patients. Understanding the molecular mechanisms that drive PND, such as the roles of neuroinflammation and mitochondrial dysfunction, can inform more targeted therapeutic strategies. Anesthetic agents with neuroprotective effects, like dexmedetomidine, could be employed to reduce postoperative cognitive dysfunction in at-risk populations. Furthermore, parallels between PND and SAE underscore the importance of addressing systemic inflammation during perioperative care. Insights from encephalocele also highlight the need to better manage postoperative complications, such as infection and CSF leakage, which may contribute to neuroinflammation and cognitive decline. Early interventions to manage oxidative stress and neuroinflammation may help prevent or mitigate cognitive decline in surgical patients. Clinicians could benefit from adopting a personalized approach to managing PND risk by taking into account factors such as the type of surgery, the anesthetic agents used, and patient-specific characteristics, including age and pre-existing conditions. Future clinical practices could incorporate biomarkers or imaging techniques to assess neuroinflammation and mitochondrial dysfunction in real time, aiding in early diagnosis and intervention.

Despite significant progress in understanding PND, several critical areas remain insufficiently explored. Future research should prioritize longitudinal studies investigating the long-term effects of anesthetic agents and their neuroprotective properties. Specifically, research should explore how different anesthetic agents interact with the mechanisms of neuroinflammation and mitochondrial dysfunction over extended postoperative periods. Additionally, further studies are needed to identify specific biomarkers for early detection of PND, which could allow for more timely and effective interventions. The parallels with SAE and encephalocele also suggest promising avenues for research into shared biomarkers, such as indicators of systemic inflammation or postoperative complications, which could be leveraged for early detection and targeted therapies. Another promising area for future investigation is the development of personalized medicine approaches for PND management. Studies exploring the genetic and molecular factors that predispose individuals to PND could provide insights into tailored anesthetic strategies and individualized therapeutic interventions. The role of microvascular changes and white matter lesions in cognitive dysfunction also warrants further exploration, as these factors may serve as important predictors of PND and help guide clinical decision-making.

## 5. Conclusions

This review underscores the multifactorial nature of PND and highlights the dual roles of anesthetic agents, which can exhibit both harmful and protective effects. Targeting critical pathways, including neuroinflammation, oxidative stress, and mitochondrial dysfunction, represents a promising strategy for mitigating PND. Insights from encephalocele and SAE emphasize both overlapping and distinct mechanisms underlying neurocognitive dysfunction, such as systemic inflammation, which may inform broader therapeutic approaches. Future research should focus on developing personalized strategies, validating biomarkers, and exploring innovative therapies to improve perioperative care and enhance cognitive outcomes in elderly surgical patients.

## Figures and Tables

**Table 1:** Mechanistic insights into postoperative neurocognitive dysfunction: Key findings and therapeutic implications.

Mechanism	Key Findings	Role of Anesthetics AND Therapeutic Implications
Neuroinflammation	Surgical and anesthetic triggers activate microglia and pathways like the NLRP3 inflammasome and NF-κB, leading to the release of pro-inflammatory cytokines such as IL-1β and TNF-α.	Dexmedetomidine reduces microglial activation and NLRP3 inflammasome activity, minimizing the release of pro-inflammatory cytokines (e.g., IL-1β, TNF-α). In contrast, sevoflurane may exacerbate inflammatory responses under certain conditions, requiring cautious use in vulnerable populations.
Neurodegeneration	Chronic neuroinflammation, oxidative stress, and mitochondrial dysfunction lead to neuronal apoptosis and synaptic loss, impairing synaptic connections and brain function.	Dexmedetomidine demonstrates neuroprotective effects by mitigating microglial overactivation and preventing mitochondrial dysfunction, thereby reducing cognitive decline. Sevoflurane may aggravate neurodegenerative processes under specific conditions and warrants careful evaluation.
Oxidative Stress	Reactive oxygen species (ROS) damage cellular components, leading to dysfunction and apoptosis.	Propofol and ketamine exhibit antioxidant effects; sevoflurane increases ROS and oxidative stress. Developing antioxidant therapies targeting ROS is essential, but further research is needed to optimize timing and dosing strategies.
Mitochondrial Dysfunction	Impaired ATP production and excessive ROS disrupt neuronal metabolism, causing apoptosis and cognitive decline.	Dexmedetomidine preserves mitochondrial integrity, while inhalational agents like isoflurane impair mitochondrial energy dynamics. Strategies include mitochondrial-targeted drugs and interventions that enhance energy metabolism to preserve neuronal function.
Neurotransmitter Imbalance	Excess glutamate triggers excitotoxicity, while reduced GABA levels fail to prevent neuronal damage.	Targeting neurotransmitter balance via NMDA receptor antagonists (e.g., ketamine) and GABA modulators reduces excitotoxicity. Antipsychotics and flumazenil may also alleviate cognitive impairment caused by imbalances.
Microvascular Changes	Blood–brain barrier (BBB) disruption and hypoperfusion exacerbate neuroinflammation and neuronal injury.	Preserving BBB integrity and enhancing microcirculation are key. Dexmedetomidine reduces BBB permeability and inflammation, sevoflurane compromises the BBB, increasing cytokine infiltration, while managing hypertension, diabetes, and oxidative stress supports vascular health and cognitive function.
White Matter Lesions	Ischemia, microvascular damage, and demyelination impair neural signaling, contributing to cognitive decline.	Therapeutic interventions focus on reducing ischemia and microvascular damage. Dexmedetomidine and other agents that support BBB integrity and vascular health may help mitigate the progression of white matter lesions.

## Data Availability

Not applicable.
